# Duration of second stage of labor and instrumental delivery as risk factors for severe perineal lacerations: population-based study

**DOI:** 10.1186/s12884-017-1251-6

**Published:** 2017-02-21

**Authors:** Marija Simic, Sven Cnattingius, Gunnar Petersson, Anna Sandström, Olof Stephansson

**Affiliations:** 1 Clinical Epidemiology Unit, T2, Department of Medicine Solna, Karolinska University Hospital and Institutet, Stockholm, SE 171 76 Sweden; 2 Department of Women’s and Children’s Health, Division of Obstetrics and Gynecology, Karolinska University Hospital and Institutet, Stockholm, SE-171 76 Sweden

**Keywords:** Pregnancy, Second stage of labor, Perineal lacerations, Instrumental delivery, Episiotomy, Occiput posterior position, Macrosomia, Partograph, Obstetric anal sphincter injury

## Abstract

**Background:**

We sought to investigate the impact of the duration of second stage of labor on risk of severe perineal lacerations (third and fourth degree).

**Methods:**

This population based cohort study was conducted in the Stockholm/Gotland region, Sweden, 2008–2014. Study population included 52 211 primiparous women undergoing vaginal delivery with cephalic presentation at term. Unconditional logistic regression analysis was used to calculate crude and adjusted odds ratios (OR), using 95% confidence intervals (CI). Main exposure was duration of second stage of labor, and main outcome was risks of severe perineal lacerations (third and fourth degree).

**Results:**

Risk of severe perineal lacerations increased with duration of second stage of labor. Compared with a second stage of labor of 1 h or less, women with a second stage of more than 2 h had an increased risk (aOR 1.42; 95% CI 1.28–1.58). Compared with non-instrumental vaginal deliveries, the risk was elevated among instrumental vaginal deliveries (aOR 2.24; 95% CI 2.07–2.42). The risk of perineal laceration increased with duration of second stage of labor until less than 3 h in both instrumental and non-instrumental vaginal deliveries, but after 3 h, the ORs did not further increase. After adjustments for potential confounders, macrosomia (birth weight > 4 500 g) and occiput posterior fetal position were risk factors of severe perineal lacerations.

**Conclusions:**

The risk of severe perineal laceration increases with duration until the third hour of second stage of labor. Instrumental delivery is the most significant risk factor for severe lacerations, followed by duration of second stage of labor, fetal size and occiput posterior fetal position.

## Background

Severe perineal laceration is a common and important complication of vaginal delivery, with a strong impact on quality of life. Depending on the anatomical structures involved, severe perineal lacerations fall into two categories: third-degree lacerations, which involve the anal sphincter complex, and fourth-degree lacerations, which extend to the rectal mucosa. Severe perineal lacerations are associated with later faecal incontinence and pelvic organ prolapse [1–3]. Third and fourth degree perineal lacerations have been reported to occur in approximately 7% of vaginal deliveries among primiparous mothers in Sweden [4]. Clinical guidelines emphasize the importance of being aware of the risk factors for obstetric anal sphincter injury in order to prevent severe perineal lacerations. Several risk factors have been identified, including primiparity, large fetal size, vaginal instrumental delivery, and occiput posterior position [5–10].

The second stage of labor is defined as the time period from complete dilation of the cervix to birth of the infant. Prolonged second stage of labor has been associated with risks of adverse maternal outcomes, including severe perineal lacerations [8, 11–16]. However, these studies have methodological limitations, including oversimplified categorization of second stage of labor, lack of information on study population characteristics and control of confounding [11, 15]. To our knowledge, no previous study has used prospectively recorded information to investigate the duration of the second stage of labor as an independent risk factor for severe perineal lacerations, taking important maternal and delivery characteristics into account.

In the present population-based cohort study, we used data recorded from the partograph to investigate the effect of duration of the second stage of labor on the risk of severe perineal lacerations in primiparous mothers.

## Methods

### Design and setting of the study

Data from the population-based Stockholm-Gotland obstetrical database were used for the study. Through the electronic medical record database, prospectively collected information was obtained from all antenatal, delivery, and postnatal care units in the regions of Stockholm and Gotland, Sweden. There were 7 delivery hospitals in the region during the study period with approximately 25 000 annual births. Detailed data on maternal, pregnancy, delivery, and infant characteristics are forwarded on a daily basis from the medical records system to the database, which contains information from 2008 onwards.

### Study population

The study population included all primiparous mothers who underwent vaginal delivery of a live singleton infant in cephalic presentation at 37 completed gestational weeks or later, between 2008 and 2014 (*n* = 52 211), which is approximately 35% of all deliveries during the study period in the region. Deliveries without partographs or notation on complete dilation of the cervix were excluded.

### Exposures and outcomes

Labor partograph data were used to measure the duration of the second stage of labor, defined as the time from the first notation of fully dilated cervix until delivery. Duration of the second stage of labor was categorised into five groups: 0 to <1 h (reference); 1 to <2 h; 2 to < 3 h; 3 to < 4 h; and ≥ 4 h.

From the first antenatal visit (usually at 7–12 gestational week), information about reproductive history, smoking habits, height, weight, state of health, family situation, the first day of the last menstrual period, were recorded by midwives. Delivery characteristics, such as onset of labor, epidural analgesia, use of oxytocin for labor augmentation, fetal head position, episiotomy, and mode of delivery were obtained from the partograph and standardized delivery records. Onset of labor was noted as either spontaneous start or induction. Use of oxytocin was analysed as any use during delivery or not. Since 2011, national guidelines with indications for augmentation with oxytocin exists. These implies use of oxytocin in the active first stage of labor when expected progress of 1 cm per hour has been delayed for 3 h or more. In the second stage, oxytocin is indicated without progress for one hour in the descending phase or 30 min or more in the pushing phase [17]. Use of methods of analgesia, including epidural, are based on the delivering mother’s preferences as well as access to different methods in the delivery hospitals [18]. Information on fetal position at delivery was recorded by midwife as occiput posterior position or not. Episiotomy included medio-lateral and midline incision. The approach to use episiotomy was decided by the obstetrician or midwife. There are no national guidelines when to use episiotomy but it is recommended to perform medio-lateral or midline incision. Mode of vaginal delivery was obtained from delivery charts and diagnostic codes according to the Swedish version of the International Classifications of Diseases, tenth revision (ICD-10), and divided into non-instrumental and instrumental vaginal deliveries (the latter included both vacuum assisted vaginal delivery and forceps delivery). Information about infant birth weight and head circumference was obtained from standard delivery charts. Fetal macrosomia was defined as birth weight of more than 4 500 grams. Hospitals included in the study, follow the recommendations on delivery management issued by Swedish Society of Obstetrics and Gynecology [18].

Gestational age was determined using the following hierarchy: a) date of embryo transfer, b) early second trimester ultrasound, which is offered to all women early in the second trimester (generally at 18 weeks), c) date of last menstrual period reported at the first antenatal visit and d) from a postnatal assessment.

The outcome measure was severe perineal laceration involving the anal sphincter (third and fourth degree). Perineal lacerations were classified according to the standardized obstetric record or diagnostic codes (ICD-10: O70.2 and O70.3).

### Statistical analyses

We investigated the effects of maternal, delivery and fetal factors on severe perineal lacerations. Unconditional logistic regression analysis was used to calculate crude and adjusted odds ratios (OR) with 95% confidence intervals (CIs). In the analysis of duration of second stage of labor and risk of severe perineal lacerations, we adjusted for maternal age, BMI, height, parental cohabitation, smoking, epidural analgesia, oxytocin augmentation, induction of labor, gestational age, episiotomy (yes/no), mode of delivery, occiput posterior position (yes/no), head circumference ≥ 35 cm (yes/no), birth weight more than 4 500 g (yes/no). Variables were categorized according to Table 1. To investigate effect modification of duration of second stage of labor by mode of vaginal delivery (non-instrumental versus instrumental) an interaction variable was included in the regression models (mode of delivery in two categories and duration of second stage of labor in five categories). Stratified analyses by mode of delivery were also performed. A *p-value* <0.05 was considered statistically significant.

**Table 1:** Maternal, fetal and delivery characteristics, among nulliparous women with singleton term vaginal births

Time^b^	Total (*n* = 52 211)	Severe perineal lacerations^a^ (*n* = 4 050)		
	n	n	%	*p-value*
0–< 1 h	16972	903	5.3	<.0001
1–< 2 h	15238	1114	7.3	
2–< 3 h	9424	849	9.0	
3–< 4 h	6217	654	10.5	
≥ 4 h	4360	530	12.2	
Maternal age				<.0001
≤ 24	8846	460	5.2	
25–29	17176	1273	7.4	
30–34	18632	1650	8.8	
≥ 35	7523	665	8.8	
Missing	34	2	-	
BMI (kg/m^2^)				0.014
< 19.9	7102	550	7.7	
19.9–24.9	30021	2271	7.6	
25.0–29.9	9099	783	8.6	
≥ 30	3021	241	7.9	
Missing	2968	205	-	
Maternal height (cm)				<.0001
130–154	1321	127	9.6	
155–159	4811	440	9.1	
160–164	12401	977	7.9	
165–169	14832	1127	7.6	
170–200	18309	1343	7.4	
Missing	537	36	-	
Cohabiting				0.9
Yes	47336	3664	7.7	
No	4385	337	7.7	
Missing	490	49		
Daily smoking				<.0001
Non-smoker	49543	3921	7.9	
Smoker	2259	106	4.7	
Missing	409	23	-	
Gestational age (weeks)				<.0001
37	2284	104	4.5	
38	5375	297	5.5	
39	11956	716	6.0	
40	16775	1351	8.1	
41	11702	1124	9.6	
42	4109	448	11.1	
Induction of labor				<.0001
Yes	8828	775	8.8	
No	43383	3275	7.5	
Epidural analgesia				< .0001
Yes	32756	2663	8.1	
No	19455	1387	7.1	
Oxytocin augmentation				< .0001
Yes	31095	2672	8.6	
No	21116	1378	6.5	
Mode of delivery				< .0001
Non-Instrumental	42261	2535	6.0	
Instrumental (vacuum extraction or forceps)	9950	1515	15.2	
Episiotomy				< .0001
Yes	4735	522	11.1	
No	47476	3528	7.4	
Occiput posterior position				< .0001
Yes	1658	229	13.8	
No	50553	3821	7.6	
Birthweight more than 4500 g				< .0001
Yes	767	161	20.9	
No	51412	3888	7.6	
Missing	32	1	-	
Head circumference more than 35.5 cm				< .0001
Yes	14613	1661	11.4	
No	37376	2366	6.3	
Missing	222	23	-	

^a^Perineal lacerations third- and fourth degree

^b^Time from fully dilated cervix to birth

The statistical software package SAS 9.4 (version 6.1; SAS, Cary, NC, USA) was used for analysis.

## Results

Among 52 211 primiparous mothers with singleton vaginal deliveries and information on the second stage of labor, 4 050 (7.8%) had third- or fourth- degree perineal lacerations.

Table 1 presents the proportion of severe perineal lacerations according to maternal, delivery, and infant characteristics. The rates of perineal lacerations increased from 5.3 to 12.2% with increasing duration of second stage of labor. Rates of perineal lacerations generally increased with maternal age, gestational age and with short maternal stature. Compared to smokers, non-smokers had higher rates of perineal lacerations. The rates of severe perineal lacerations in women with vaginal instrumental and vaginal non-instrumental deliveries were 15.3% and 6.0%, respectively. Vacuum extraction was the most common method used in 3 940 deliveries while forceps were used in 110 (2.7%) instrumental deliveries. Occiput posterior position, a high birth weight and a large head circumference were also associated with increased rates of perineal lacerations (13.8%, 20.9% and 11.4% respectively).

Rates by mode of delivery are presented graphically in Table 1. In instrumental vaginal deliveries, rates of severe perineal lacerations were much higher at each time point than in spontaneous vaginal deliveries. In both groups, the rates remained about the same beyond the third hour.

When we calculated the risk of third and fourth degree lacerations depending on mode of vaginal delivery, we adjusted for duration of second stage of labor, maternal age, BMI, maternal height, cohabitation, smoking, epidural analgesia, oxytocin augmentation, induction of labor, gestational age, episiotomy, occiput posterior position, head circumference and birth weight. The aOR for instrumental deliveries was 2.24, 95% CI 2.07–2.42 (crude OR 2.87, 95% CI 2.68–3.07).

The results of the crude and adjusted logistic regression analyses of duration of second stage of labor and severe perineal lacerations are presented in Table 2. Compared to women with duration of second stage of labor less than 1 h, the adjusted OR for perineal lacerations for 2- < 3 h was 1.42; 95% CI 1.28–1.58. Similar risks were found for second stage of labor of 3- < 4 h: OR 1.45; 95% CI 1.29–1.64; and >4 h: OR 1.41, 95% CI 1.24–1.61.

**Table 2:** Risk of severe perineal lacerations among nulliparous by duration of second stage of labor

Time^c^			Severe perineal lacerations^a^			
			Crude		Adjusted^b^	
	N	%	OR	95% CI	aOR	95% CI
0–< 1 h	16972	5.3	1.00	Ref	1.00	Ref
1–< 2 h	15238	7.3	1.40	1.28–1.54	1.25	1.13–1.38
2–< 3 h	9424	9.0	1.76	1.59–1.94	1.42	1.28–1.58
3–< 4 h	6217	10.5	2.09	1.88–2.32	1.45	1.29–1.64
≥4 h	4368	12.1	2.46	2.20–2.76	1.41	1.24–1.61

^a^Perineal lacerations third and fourth degree.^b^Adjusted for maternal age, BMI, maternal height, parental cohabitation, smoking, epidural analgesia, oxytocin augmentation, induction of labor, gestational age, episiotomy, occiput posterior position, head circumference, birthweight more than 4500 g and mode of delivery

^c^Time from fully dilated cervix to birth

In stratified analyses by mode of vaginal delivery, rates and adjusted OR are presented in Table 3. In instrumental vaginal deliveries, rates of severe perineal lacerations were higher at each time point than in spontaneous vaginal deliveries but remained the same after 2 h.

**Table 3:** Risk of severe perineal lacerations, maternal, fetal and delivery characteristics by mode of vaginal delivery

Time^c^	Severe perineal lacerations^a^							
	Instrumental delivery				Non- instrumental delivery			
	n	%	aOR^b^	95% CI	n	%	aOR^b^	95% CI
0–< 1 h	1507	10.9	1.00	Ref	25465	4.8	1.00	Ref
1–< 2 h	2042	14.7	1.38	1.11–1.71	13196	6.2	1.21	1.08–1.35
2–< 3 h	2066	16.3	1.62	1.31–1.99	7358	6.9	1.34	1.18–1.53
3–< 4 h	2164	16.6	1.61	1.32–2.02	4053	7.3	1.39	1.20–1.63
≥ 4 h	2171	16.3	1.51	1.22–1.86	2189	8.1	1.45	1.19–1.75
Maternal age								
< 24	1123	11.2	0.86	0.70–1.06	7723	18.3	0.73	0.63–0.84
25–30	3044	30.3	1.00	Ref	14132	33.6	1.00	Ref.
30–35	3918	39.0	1.05	0.91–1.21	14714	34.9	1.22	1.11–1.36
> 35	1953	19.5	0.95	0.80–1.12	5570	13.2	1.17	1.02–1.33
BMI (kg/m2)								
< 19.9	1329	14.1	1.16	0.98–1.38	5773	14.5	1.07	0.95–1.21
20–24.9	5718	60.6	1.00	Ref.	24303	61.1	1.00	Ref.
25–29.9	1834	19.4	1.18	1.02–1.36	7265	18.2	1.10	0.95–1.21
> 30	561	5.9	0.99	0.77–1.27	2460	6.2	1.18	0.99–1.40
Maternal height								
130–154	325	3.3	1.44	1.05–1.97	996	2.4	1.48	1.13–1.95
155–159	1097	11.1	1.29	1.06–1.57	3714	8.9	1.42	1.22–1.66
160–164	2524	25.4	0.99	0.84–1.16	9877	23.7	1.15	1.03–1.30
165–169	2845	28.6	1.00	Ref	11987	28.7	1.00	Ref.
170–200	3154	31.7	0.83	0.71–0.96	15155	36.3	0.95	0.86–1.06
Daily smoking								
Non-smoker	9631	96.5	1.00	Ref	39912	95.4	1.00	Ref.
smoker	352	3.5	0.89	0.64–1.24	1907	4.5	0.64	0.49–0.84
Gestational age (weeks)								
37	339	9.1	0.59	0.39–0.88	1945	3.7	0.64	0.49–0.82
38	740	11.6	0.75	0.58–0.97	4365	4.5	0.75	0.64–0.88
39	1878	12.1	0.76	0.63–0.91	10078	4.8	0.80	0.71–0.90
40	3158	15.5	1.00	Ref	13617	6.3	1.00	Ref.
41	2617	17.3	1.09	0.94–1.26	9085	7.4	1.09	0.98–1.23
42	1218	18.7	1.14	0.93–1.11	2901	7.9	1.14	0.96–1.35
Induction of labor	2204	16.3	0.95	0.82–1.11	6624	6.3	1.04	0.92–1.18
Epidural analgesia	7574	15.3	0.94	0.82–1.08	25182	5.9	0.88	0.80–0.97
Oxytocin augmentation	7998	15.4	1.01	0.87–1.18	23097	6.3	0.95	0.87–1.05
Occiput posterior position	685	20.7	1.89	1.54–2.32	973	8.9	1.45	1.13–1.85
Episiotomy	1893	13.9	0.81	0.70–0.94	28442	9.1	1.46	1.27–1.69
Birthweight > 4500 g	242	31.4	2.22	1.65–2.99	525	16.2	2.27	1.77–2.93
Head circumference >35.5 cm	3970	18.8	1.48	1.31–1.67	10643	8.6	1.51	1.37–1.66

^a^Perineal lacerations third- and fourth degree

^b^Adjusted for maternal age, BMI, maternal height, parental cohabitation, smoking, epidural analgesia, oxytocin augmentation, induction of labor, gestational age, episiotomy, occiput posterior position, head circumference, birthweight more than 4500 g and mode of delivery

^c^Time from fully dilated cervix to birth

The relative risk of perineal laceration increased with duration of second stage of labor during the first 3 h but then remained almost unaltered, both in instrumental and non-instrumental deliveries. The absolute risk of severe perineal lacerations was more than 2 times higher in instrumental versus non-instrumental deliveries for each category of second stage duration. The occiput posterior fetal head position, high birth weight and large head circumference were significantly associated with perineal lacerations in both vaginal instrumental and non-instrumental deliveries. Episiotomy was a significant protector for severe perineal lacerations among vaginal instrumental deliveries, whereas it was a significant risk factor among non-instrumental deliveries (OR 0.81, 95% CI 0.70–0.94 and OR 1.46, 95% CI 1.27–1.69, respectively). (Table 3) The test of interaction for duration of second stage of labor and mode of delivery was not significant (*p* = 0.43).

## Discussion

In this large population-based cohort study we found an increasing risk of severe perineal lacerations within first 3 h of second stage of labor. Instrumental delivery was the most important risk factor for severe lacerations, together with parameters indicating large fetal size, such as high birth weight and large head circumference, and occiput posterior fetal position.

We used a population-based cohort from an electronic perinatal database based on medical records with prospectively recorded information analysed retrospectively. This enabled us to investigate the length of the second stage of labor in relation to severe perineal lacerations in various clinical scenarios. Our study base included a large population of primiparous women with detailed information on demographic data, maternal and delivery characteristic, which made it possible to control for relevant confounders.

Patients were recruited over a 6-year period, reflecting current practice in the study region between 2008 and 2014. The study period was short, which precluded any examination of labor patterns over time. Management of labor at the seven hospitals in the study region may differ from that of other regions, thereby limiting the generalizability of our findings. However, the majority of clinics involved in the study follow the current management recommendations issued by the Swedish Society of Obstetricians and Gynecologists, which are based on international guidelines [18].

The prevalence of third and fourth degree lacerations (7.8%) was similar to a previously reported prevalence for the Stockholm-Gotland region (8.2%) but higher than the national Swedish prevalence (6.6%) [4, 19]. This can be explained by differences in population characteristics between the Stockholm-Gotland region (predominantly a large city region) and the rest of the country. Furthermore, the diagnosis of lacerations is based on clinical examination, and classification of the degree of perineal laceration could vary between hospitals and regions. Previously, it has been described that cases of clinically undetected sphincter lacerations have later been diagnosed by endo-anal ultrasound, which indicates that the incidence of severe perineal injures, may be higher than reported [20]. Still, we have no reason to believe that diagnostic accuracy would vary by length of second stage of labor, and any possible misclassification would be non-differential.

Duration of the second stage of labor was defined as the time from complete dilatation of the cervix until birth based on the first notation of a fully dilated cervix. The transition between the first and second stages of labor could not be precisely established, and recorded data depended on the timing of cervical examination relative to time of complete cervical dilation. Although previous studies have focused on adverse maternal and perinatal outcomes associated with prolonged duration of the second stage of labor, several studies have not observed an increased risk of severe perineal lacerations in women with prolonged second stage of labor [6, 8, 14]. However, in a cohort of women with term deliveries, rates of perineal lacerations increased with increasing duration of labor, after controlling for potential confounding variables [11, 13, 15]. Cheng et al., showed that a prolonged second stage of labor increased the risk of severe perineal lacerations after adjusting for maternal, fetal and delivery characteristics, and for instrumental vaginal delivery [11]. Rousse et al. also showed that risk for severe perineal lacerations increased for each additional hour of the second stage, after adjusting for mode of delivery [15]. Our results correspond well with these findings, although the risk estimation in our study was of lower magnitude. One possible explanation is that we included additional factors in the analysis, such as fetal head position and fetal head circumference.

Mode of vaginal delivery (non-instrumental versus instrumental) has been identified as an effect modifier in studies evaluating the influence of duration of the second stage on risks of severe perineal lacerations [8, 15]. Given the causal relationship between duration of the second stage and instrumental delivery, we wanted to investigate the independent effect of duration of the second stage of labor in instrumental and non-instrumental vaginal deliveries in stratified analyses. We found that the risk for perineal lacerations increased during the first 3 h and thereafter remained relatively unaltered in both instrumental and non-instrumental deliveries. Similarly to our results, Cheng et al. reported increased risk (OR 1.35) of perineal lacerations after instrumental delivery beyond 3 h of second stage [21]. However, the risk was not calculated for each additional hour of second stage of labor.

In accordance with our results, it has been demonstrated that large head circumference and higher birth weight increase the risk of perineal lacerations [22].

In previous research, epidural use has been associated with both a protective effect and increased risk of lacerations. Thus, we considered epidural analgesia to be a potential confounder, which we adjusted for in the multivariable model [23–25].

Although literature agrees that routine midline episiotomy is associated with increased rates of severe perineal lacerations [17], recent studies suggest that use of medio lateral episiotomy protects against these injuries during instrumental delivery [26, 27]. Our results, similar to previously reported, emphasize that episiotomy may be a risk factor for perineal lacerations in non-instrumental vaginal deliveries, but reduces the risk in vaginal instrumental deliveries [28]. There is no reason to believe that this difference is caused by the type of episiotomy used, since the most usual used is medio lateral episiotomy.

Besides increased risk of severe perineal lacerations, a prolonged second stage of labor is also associated with other adverse maternal outcomes such as postpartum haemorrhage, fever, infection and urinary retention [11–16]. The question when to intervene should involve thorough evaluation of the ongoing risks from further expectant management.

## Conclusion

We conclude that the risk of severe perineal lacerations increases during the first 3 hours of second stage of labor and thereafter remains relatively unchanged. Risk factors for perineal lacerations were instrumental delivery, large fetal size and occiput posterior position. This important information should be considered when weighting the risks and benefits of performing instrumental delivery, especially if the goal is to reduce the risk of maternal perineal trauma. We suggest that the decision to perform an instrumental vaginal delivery should be weighed against the option of continuing labor in the setting of reassuring maternal and fetal status. Unless there are signs of fetal distress or strong maternal discomfort, continuing labor may enable significant proportion of women to achieve spontaneous vaginal delivery, and thus lead to decrease in severe perineal lacerations.

## Abbreviations

CI: Confidence interval
ICD: the International Classifications of Diseases
OR: Odds ratios
